# Polypharmacy in patients with multiple sclerosis and the impact on levels of care and therapy units

**DOI:** 10.3389/fneur.2023.1330066

**Published:** 2023-12-21

**Authors:** Finn Brüggemann, Stefan Gross, Marie Süße, Pavel Hok, Sebastian Strauss, Tjalf Ziemssen, Niklas Frahm, Uwe K. Zettl, Matthias Grothe

**Affiliations:** ^1^Department of Neurology, University Medicine Greifswald, Greifswald, Germany; ^2^Department of Internal Medicine B, University Medicine Greifswald, Greifswald, Germany; ^3^DZHK (German Center for Cardiovascular Research), Partner Site Greifswald, Greifswald, Germany; ^4^MS Center, Center of Clinical Neuroscience, University Clinic Carl-Gustav Carus, Dresden University of Technology, Dresden, Germany; ^5^Department of Neurology, University Medicine Rostock, Rostock, Germany

**Keywords:** multiple sclerosis, polypharmacy, health costs, level of care, comorbidity

## Abstract

**Background:**

The aim of this study was to examine the societal costs of polypharmacy in patients with multiple sclerosis (MS). We therefore focused on the association between the number of medications on the level of care (LOC), the German classification of the need for care, and the number of therapy sessions (TTU).

**Methods:**

In addition to demographic information and medication, 101 MS patients performed the Multiple Sclerosis Health Resource Utilization Survey (MS-HRS). Medications were subdivided into a total number of medications (TD), MS-related medication [MSD, i.e., disease-modifying drugs (DMDs) and symptomatic treatment (SD)], and medication for comorbidities (CDs). Multivariate linear regression models were performed to estimate if the amount of each medication type affects LOC or TTU.

**Results:**

Polypharmacy appeared in 54 patients at the time of the survey. The relative risk (RR) of LOC 1 increased significantly by 2.46 (*p* = 0.001) per TD and by 2.55 (*p* = 0.004) per MSD, but not per CD (RR 1.44; *p* = 0.092). The effect of RR on MSD was driven by SD (RR 2.2; *p* = 0.013) but not DMD (RR 2.6; *p* = 0.4). RR of MSD remained significant for LOC 2 (1.77; *p* = 0.009) and LOC 3/4 (1.91; *p* = 0.015), with a strong trend in RR of SD, but not DMD. TTU increased significantly per MSD (*p* = 0.012), but not per TD (*p* = 0.081) and CD (*p* = 0.724).

**Conclusion:**

The number of MSDs is related to the likelihood of a higher level of care and the number of therapy sessions and is therefore a good indication of the extent of the societal costs.

## Introduction

Multiple sclerosis (MS) is a chronic inflammatory disease of the central nervous system characterized by inflammation, demyelination, axonal damage, and degeneration (1, 2). MS currently affects approximately 2.8 million people worldwide, with an ongoing increase in incidence and prevalence (3). The disease-related progression is reduced (4) and the life expectancy of MS patients has improved (5) in the past years due to disease-modifying drugs (DMDs) (6).

In addition to DMD decreasing disease activity, persisting symptoms of MS such as pain (7), paresthesias, coordination disorders (8), spasticity (9), fatigue (10), and depression (11) also often necessitate symptomatic drug treatment (SD) or non-drug treatments such as physiotherapy and occupational therapy (12).

Comorbidities and their need for specific drug therapy (CD) are the third component in the therapeutic management of MS, which becomes more likely with the increasing age of the patients (13–15). Individual treatment regimens with DMDs, SDs, and CDs often result in polypharmacy, which is commonly defined as the use of ≥5 medications (16–19). The prevalence of polypharmacy in MS and its impact on disease progression have become a focus of interest in recent years (20–26). Polypharmacy in the elderly is associated with an increased risk of negative health outcomes such as drug-associated side effects, drug interactions, reduced therapy adherence, and rehospitalization (27, 28).

In addition to these medical issues, multimodal treatment strategies also lead to a considerable socioeconomic burden on the healthcare system. Disease-related costs can be divided into direct costs, e.g., due to medication and hospitalization, and indirect costs, e.g., due to the loss of ability to work, with all costs associated with disease-related disability increasing in MS (29–31). The average annual societal cost per patient in Europe is 40.000€, but recent German data reveal that non-medical direct costs such as community services and informal care, as well as indirect costs due to long-term absence, invalidity, and early retirement, are the main causes of increased costs, especially for patients with a higher degree of disability (8).

Cumulative disability in MS patients also leads to an increasing dependence on family or caregivers and an increased need for care requirements (8). In Germany, the costs are partly financed via the respective level of care (LOC). The LOC is used in Germany to classify the need for care. It assesses impairments in independence and abilities in the areas of mobility, communication, and activities of daily living, as well as impairments due to illness and therapy. It is divided into grades from 1 (slight impairment of independence or abilities) to 5 (most severe impairment of independence or abilities with special requirements for nursing care). Financial support in Germany is provided depending on these LOC (32).

The link between polypharmacy itself and health-related societal costs has been demonstrated in the elderly (27, 28), but such data are lacking in MS. Therefore, the primary aim of our study was to investigate the association between polypharmacy and the LOC as an economical index in MS. As a secondary aim, we hypothesized that the number of drugs is also related to the amount of physiotherapy and occupational therapy, as assessed by the number of therapeutic treatment units (TTUs). To further explain these possible associations, we carried out these calculations for all types of medications [i.e., the total amount of drugs (TD), DMD, SD, and CD].

## Materials and methods

The prospective cohort study was approved by the Local Ethics Committee of the University Medical Center Greifswald (BB137/21) and was conducted in accordance with the Declaration of Helsinki. The survey of patients took place between June 2021 and June 2022 at the University Medical Center Greifswald during an outpatient or inpatient stay. The inclusion criteria were a diagnosis of MS according to the 2017 revised McDonald criteria (33). A structured patient interview including sociodemographic, clinical, and neurological aspects and a detailed medication history was conducted. In addition, patients completed the Multiple Sclerosis Health Resource Utilization Survey (MS-HRS) to assess economic impact (28). Sociodemographic data included sex, age, and years of education. Clinical neurological data included disease course, disease duration, and level of disability using the Expanded Disability Status Scale (EDSS) (34). In addition, the number of comorbidities and the number of medications were recorded. Medications were divided into TD, MS-related drugs (MSDs), and medications for comorbidities (CDs). MSD summarizes DMD and SD.

We divided the patients into groups with (PwP) and without polypharmacy (Pw/oP) according to the most common definition as the concurrent use of ≥5 medications (15–18).

The MS-HRS is used to estimate the economic impact of MS. The MS-HRS is a validated survey covering both direct and indirect costs based on the patients' statements over the last 6 months at the time of the survey (28). For our analysis, we focused on the parameters LOC and TTU. Visits to physiotherapists and occupational therapists were combined under TTU.

## Statistical analysis

For baseline patient characteristics, we present continuous variables as mean value (standard deviation: SD) and categorical variables as absolute number (percentage) if not otherwise stated.

To analyze LOC as an outcome, we used multivariable multinomial regression models adjusted for the number of comorbidities, EDSS, age, and current smoking status to calculate relative risk ratios (RRRs) to indicate the change in the relative risk (RR) of being in LOC 1, 2, or 3/4 vs. “no care” as a reference outcome category per intake of one additional respective medication. We combined LOC 3 and LOC 4 as groups because LOC 4 occurred in only one patient in our cohort. In addition to MSD, we also evaluated both components, DMD and SD, separately.

To analyze the potential association between polypharmacy (number of respective medications) and TTU as an outcome, we used multivariable linear regression models adjusted for the number of comorbidities, EDSS, age, and current smoking status.

## Results

### Patient characteristics

In our study, 101 MS patients (78 women, 23 men) were included (see Table 1). The mean age at baseline was 49.8 years (SD: 12.41), and the disease duration at baseline was 12.4 years (SD: 9.22). The median EDSS score was 4 (range 0–8). While 80 patients had at least one comorbidity, 21 had none. The mean number of comorbidities was 1.94 (SD: 1.65). Polypharmacy was present in 54 patients. The mean number of medications was 5.25 (SD: 2.9) for TD, 2.56 (SD: 1.77) for MSD, and 2.76 (SD: 2.55) for CD. Five patients had a LOC 1, 17 patients had a LOC 2, and 10 patients had a LOC 3. One patient had a LOC 4, and LOC 5 did not occur in our cohort. No LOC was present in 35 patients. The mean number of TTUs in the last 6 months was 27.56 (SD: 27.39). Many patients had a standing prescription and, in some cases, up to several units of therapy per week (for details, see Table 2).

**Table 1:** Patients' characteristics.

	***n* (%)**	**Mean**	**SD**
Patients	101		
Female	78 (77.2)		
Male	23 (22.8)		
Age at baseline (y)		49.8	12.41
Disease duration at baseline (y)		12.4	9.22
RRMS	68 (67.3)		
SPMS	22 (21.8)		
PPMS	11 (10.9)		
EDSS (median/range)		4	0–8
Current smoking (yes)	33 (32.7)		
Comorbidities		1.94	1.65
PwC	80 (79.2)		
Pw/oC	21 (20.8)		
PwP	54 (53.5)		
Pw/oP	47 (46.5)		

RRMS, relapsing-remitting multiple sclerosis; SPMS, secondary progressive multiple sclerosis; PPMS, primary progressive multiple sclerosis; EDSS, expanded disability status scale; PwC, patients with comorbidity; Pw/oC, patients without comorbidity; PwP, patients with polypharmacy; Pw/oP, patients without polypharmacy.

**Table 2:** Quantity of medication, level of care, and therapy units.

	***n* (%)**	**Mean**	**SD**
Total medication		5.25	2.9
MS medication (i.e., DMD + SD)		2.56	1.77
SD		1.76	1.72
PwDMD	78 (77.2)		
Pw/oDMD	23 (22.8)		
Comorbidity medication		2.76	2.55
no LOC	69 (68.3)		
LOC 1	5 (5.0)		
LOC 2	16 (15.8)		
LOC 3	10 (9.9)		
LOC 4	1 (1)		
LOC 5	0 (0)		
Therapie units		27.56	27.39
TTU 0	35 (34.7)		
TTU 1–20	10 (9.9)		
TTU 21–40	17 (16.8)		
TTU 41–60	26 (25.7)		
TTU 61–80	10 (9.9)		
TTU >81	3 (3.0)		

SD, symptomatic treatment; DMD, disease-modifying drugs; PwDMD, patients with DMD; Pw/oDMD, patients without DMD; LOC, level of care; TTU, therapy units (last 6 months).

### Regression models

The relative risk (RR) of being in LOC 1 vs. “no care” (reference) increased significantly by a relative risk ratio (RRR) of 2.46 (95% CI: 1.46–4.16; *p* = 0.001) per one additional TD and by RRR = 2.55 (1.34–4.85; *p* = 0.004) per one additional MSD. As a sensitivity analysis, we also investigated DMD and SD separately: RRR = 2.62 (0.29–23.16; *p* = 0.385) for DMD and RRR = 2.20 (1.18–4.10; *p* = 0.013) for SD. Thus, the effect of MSD is mainly driven by SD. In contrast, the relative risk ratio was not significantly increased for CD (RRR = 1.44; 0.94–2.19; *p* = 0.092).

**Figure 1:**
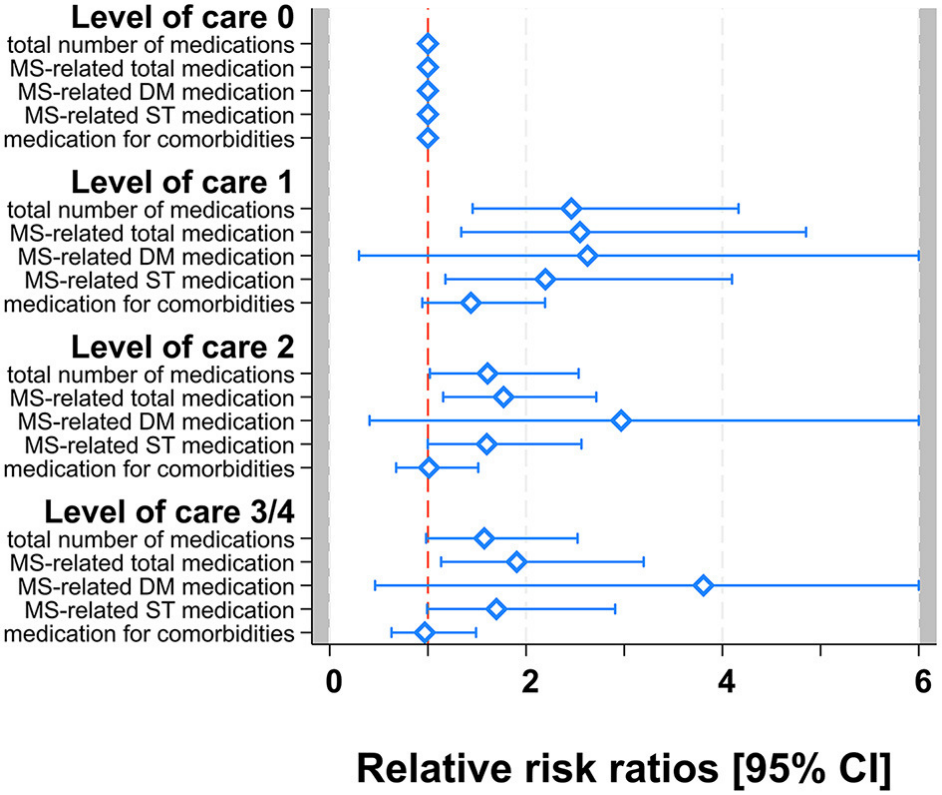
Multivariable multinomial models for TD, MSD [DMD, SD], and CD. Forest-plot of relative risk ratios with 95% confidence limits. Zoom-in with RRR scale truncated at 6 for better visibility.

Furthermore, the relative risk ratio of MSD remained significant also for LOC 2 (RRR = 1.77; 1.16–2.72; *p* = 0.009; DMD: RRR = 2.97; 0.41–21.75; *p* = 0.284; SD: RRR = 1.60; 1.00–2.56; *p* = 0.051) and LOC 3/4 (RRR = 1.91; 1.13–3.20; *p* = 0.015; DMD: RRR = 3.81; 0.46–31.40; *p* = 0.214; SD: RRR = 1.70; 0.99–2.91; *p* = 0.054), whereas RRR of TD was significantly increased for LOC 2 (RRR = 1.61; 1.02–2.54; *p* = 0.041) but non-significantly for LOC 3/4 (RRR = 1.57; 0.98–2.52; *p* = 0.06). The relative risk ratios for CD remained non-significant as well for the higher LOCs (LOC 2: RRR = 1.01; 0.68–1.51; *p* = 0.957; LOC 3/4: RRR = 0.97; 0.63–1.49; *p* = 0.883) (see Figure 1 and Table 3).

**Table 3:** Relative risk ratio of level of care vs. “no care” by one additional medication in different medication groups.

**Level of care**	**Medication**	**RRR**	**95%CI**	***p***
LOC 1	Total medication	2.46	1.46–4.16	0.001
	MS medication	2.55	1.34–4.85	0.004
	DMD	2.62	0.29–23.16	0.385
	SD	2.2	1.18–4.10	0.013
	Comorbidity medication	1.44	0.94–2.19	0.092
LOC 2	Total medication	1.61	1.02–2.54	0.041
	MS medication	1.77	1.16–2.72	0.009
	DMD	2.97	0.41–21.75	0.395
	SD	1.6	1.00–2.56	0.051
	Comorbidity medication	1.01	0.68–151	0.957
LOC 3/4	Total medication	1.57	0.98–2.52	0.06
	MS medication	1.91	1.13–3.20	0.015
	DMD	3.81	0.46–31.4	0.214
	SD	1.7	0.99–2.91	0.054
	Comorbidity medication	0.97	0.63–1.49	0.883

RRR, relative risk ratio; CI, confidence interval; LOC, level of care; MS, multiple sclerosis; DMD, disease-modifying drugs; SD, symptomatic drug treatment.

TTU within the last 6 months increased significantly per one additional MSD (β = 3.02; 95%CI: 0.67–5.38; *p* = 0.012; DMD: β = 5.22; −4.64–15.07; *p* = 0.296; SD: β = 2.87; 0.26–5.49; *p* = 0.031), but not per increase in TD (β = 0.81; −1.02–2.64; *p* = 0.081) or CD (β = −0.49; −3.26–2.27; *p* = 0.724).

## Discussion

In this study, for the first time, we systematically examined the impact of the number of medications on relevant socio-economic aspects. We demonstrated that the number of medications was associated with both LOC and TTU in our MS cohort. As both variables are indicators of increased symptomatology relevant to the financial burden on the healthcare system, we were able to confirm the association between polypharmacy in MS and the socio-economic burden of the disease.

The proportion of PwP in our real-life cohort was 53.5%. These data are in line with the few studies focusing on polypharmacy in MS, especially in comparable German cohorts (20, 35). The high amount of polypharmacy highlights the importance of further research, especially as the mean age of the patients will increase further in future, also leading to an increase in comorbidity and polypharmacy (36).

In Germany, the level of care is used to classify the financial need for care. The assessment is based on a point system and evaluates various components such as mobility, cognition, communication, self-care, dealing with disease- and therapy-related requirements, and everyday life (32). The degree of disability and the severity of the symptoms are determining factors for the LOC classification in relation to MS. The amount of the monthly care allowance depends on the degree of LOC, and a distinction is also made between outpatient and inpatient care (37). TTU can also result in significant costs for the healthcare system, particularly in the case of permanent prescriptions (29).

There was a significantly higher risk of being at LOC 1 or 2, but not 3/4, with an increased number of drugs taken. This increasing risk was significant for each LOC for the number of MSDs but not for the CD, which suggests that the increase in LOC is primarily driven by MS medications, i.e., DMD and SD. The number of TTUs also showed a significant factorial increase only with the increasing number of MSD, but not the TD or CD. Thus, the association of both the LOC and the TTU is driven by the number of MS-related drugs, despite adjusting the regression for possible explaining confounders such as EDSS, number of comorbidities, age of the patients, and current smoking status. We are not aware of any study investigating the effect of polypharmacy on LOC or TTU so far, even in other diseases.

The increase in MSD is mainly due to SD, as DMDs are used as an immunological monotherapy therapy for MS. An exception is a current relapse of MS, in which corticosteroids may be used in addition to DMD. This was the case in three patients (3.0%) in our study. In total, 75 patients (74.3%) were taking a DMD, while 23 patients (22.8%) were not taking any DMD. Whether and how many SDs are taken depends largely on individual disease severity and symptomatology. With a higher degree of clinical symptoms, symptomatic treatment is usually a combination of medical and non-medical approaches, such as physical therapy and occupational therapy. A study of the German MS Registry showed that motor symptoms such as impaired walking, spasticity, and ataxia/tremor are often treated multimodally with drugs in combination with non-drug strategies (38). This could also explain the association between MSD and TTU. This hypothesis is also supported by the fact that there is no significant correlation between TD and especially CD and TTU, but more data are needed to rule out this possible interaction.

The association between MSD and LOC might also be due to the fact that symptoms of MS such as motor or sensory impairments more often result in a higher LOC classification than symptoms of comorbidities such as hyperlipidemia or hypertension (14). Because the number of patients at each LOC stage in our cohort was quite small, studies with larger cohorts are needed to better demonstrate the impact.

Although the regression was adjusted for EDSS, we are aware that especially the amount of SD is correlating with the severity of the symptoms and therefore the EDSS. On the other hand, the significant correlation even with the statistical adjustment reveals that the number of MS-related medications provides additional information about the level of care in patients with the same EDSS.

The study has several limitations. First, our data were collected at a single time point. We were interested in the association between polypharmacy and health economic variables, but especially longitudinal studies are needed in future. Furthermore, we did not use the exact direct and indirect costs but rather used a survey to quantify economic variables such as LOC and TTU. We chose the MS-HRS, as it is a widely used survey validated in a German cohort, but we are aware that these measurements represent only some aspects of the financial burden.

In conclusion, this study shows that polypharmacy is an important component to consider for MS patients and the healthcare system. We found that an increased number of medications leads to a significantly increased relative risk of higher LOC, which is driven by MS-specific drugs. The increasing number of MS-specific drugs was also associated with an increased TTU within the last 6 months. Polypharmacy, especially for MS-related medications, is therefore a good indicator of the burden on the healthcare system for MS patients.

## Data availability statement

The raw data supporting the conclusions of this article will be made available by the authors, without undue reservation.

## Ethics statement

The study was approved by University Medical Center Greifswald (BB137/21). The study was conducted in accordance with the local legislation and institutional requirements. All participants provided their written informed consent to participate in this study.

## Author contributions

FB: Writing – original draft, Data curation. SG: Formal analysis, Visualization, Writing – review & editing. MS: Writing – review & editing. PH: Writing – review & editing. SS: Writing – review & editing. TZ: Writing – review & editing. NF: Writing – review & editing. UZ: Writing – review & editing. MG: Methodology, Supervision, Writing – review & editing.
